# Exacerbations in patients with chronic obstructive pulmonary disease receiving physical therapy: a cohort-nested randomised controlled trial

**DOI:** 10.1186/1471-2466-14-71

**Published:** 2014-04-26

**Authors:** Emmylou Beekman, Ilse Mesters, Erik JM Hendriks, Jean WM Muris, Geertjan Wesseling, Silvia MAA Evers, Guus M Asijee, Annemieke Fastenau, Hannah N Hoffenkamp, Rik Gosselink, Onno CP van Schayck, Rob A de Bie

**Affiliations:** 1 Department of Epidemiology, CAPHRI School for Public Health and Primary Care, Maastricht University, PO Box 616, Maastricht, MD 6200, The Netherlands; 2 Centre for Evidence Based Physiotherapy, Maastricht University, Maastricht, The Netherlands; 3 Physical therapy practice in multidisciplinary centre, ParaMedisch Centrum Sittard Zuid, Sittard, The Netherlands; 4 Physical therapy practice, Fysiotherapie Maasstaete, Druten, The Netherlands; 5 Department of Family Medicine, CAPHRI School for Public Health and Primary Care, Maastricht University, Maastricht, The Netherlands; 6 Department of Respiratory Medicine, CAPHRI School for Public Health and Primary Care, Maastricht University Medical Centre, Maastricht, The Netherlands; 7 Department of Health Services Research, CAPHRI School for Public Health and Primary Care, Maastricht University, Maastricht, The Netherlands; 8 Department of Public Mental Health, Trimbos Institute, Netherlands Institute of Mental Health and Addiction, Utrecht, The Netherlands; 9 Boehringer Ingelheim, Alkmaar, The Netherlands; 10 International Victimology Institute Tilburg, Tilburg University, Tilburg, The Netherlands; 11 Department of Rehabilitation Sciences, KU Leuven, Leuven, Belgium; 12 Department of Respiratory Rehabilitation and Respiratory Division, University Hospital Leuven, Leuven, Belgium

**Keywords:** Chronic obstructive pulmonary disease (MESH), Disease exacerbation (MESH), Physical therapy modalities (MESH), Physiotherapy, Exercise therapy (MESH), Pulmonary rehabilitation, Costs and cost analysis (MESH), Quality of life (MESH), Comorbidity (MESH), Cohort studies (MESH), Randomized controlled trial (MESH)

## Abstract

**Background:**

Physical exercise training aims at reducing disease-specific impairments and improving quality of life in patients with chronic obstructive pulmonary disease (COPD). COPD exacerbations in particular negatively impact COPD progression. Physical therapy intervention seems indicated to influence exacerbations and their consequences. However, information on the effect of physical therapy on exacerbation occurrence is scarce. This study aims to investigate the potential of a protocol-directed physical therapy programme as a means to prevent or postpone exacerbations, to shorten the duration or to decrease the severity of exacerbations in patients with COPD who have recently experienced an exacerbation. Besides, this study focuses on the effect of protocol-directed physical therapy on health status and quality of life and on cost-effectiveness and cost-utility in patients with COPD who have recently experienced an exacerbation.

**Methods/Design:**

A prospective cohort of 300 COPD patients in all GOLD stages will be constructed. Patients will receive usual multidisciplinary COPD care including guideline-directed physical therapy. Patients in this cohort who have GOLD stage 2 to 4 (post-bronchodilator FEV_1_/FVC < 0.7 and FEV_1_ < 80% of predicted), who receive reimbursement by health insurance companies for physical therapy (post-bronchodilator Tiffeneau-index < 0.6) and who experience a COPD exacerbation will be asked within 56 days to participate in a cohort-nested prospective randomised controlled trial (RCT). In this RCT, the intervention group will receive a strict physical therapy programme for patients with COPD. This protocol-directed physical therapy (pdPT) will be compared to a control group that will receive sham-treatment, meaning no or very low-intensity exercise training (ST). An economic evaluation will be embedded in the RCT. Anthropometric measurements, comorbidities, smoking, functional exercise capacity, peripheral muscle strength, physical activity level, health related quality of life, patients’ perceived benefit, physical therapy compliance, motivation level, level of effective mucus clearance, exacerbation symptoms and health care contacts due to COPD will be recorded. Follow-up measurements are scheduled at 3 and 6 weeks, 3, 6, 12 and 24 months after inclusion.

**Discussion:**

Ways to minimise potential problems regarding the execution of this study will be discussed.

**Trial registration:**

The Netherlands National Trial Register NTR1972.

## Background

Chronic obstructive pulmonary disease (COPD) is currently defined as “a common preventable and treatable disease, characterised by persistent airflow limitation that is usually progressive and associated with an enhanced chronic inflammatory response in the airways and the lung to noxious particles or gasses. Exacerbations and comorbidities contribute to the overall severity in individual patients” [1]. The World Health Organisation (WHO) lists COPD as the tenth most prevalent disease worldwide and the fourth most common cause of death in the world, responsible for 5% of overall mortality [2]. Due to the ageing population, expanding smoking behaviour, earlier diagnosis of COPD and reduced mortality from other common causes of death, the total number of people with COPD will increase in the near future. This will rank COPD fifth worldwide in burden of disease by 2020 [1].

Common clinical pulmonary manifestations that can be seen in COPD patients are dyspnoea with chronic cough, sputum production and recurrent respiratory infections. Additionally, with disease progression significant extrapulmonary systemic effects can be observed in patients, especially in patients with moderate to severe airway obstruction: skeletal muscle dysfunction and weakness, nutritional abnormalities and weight loss [1,3]. Nowadays, systemic effects of COPD are acknowledged as an important characteristic of the disease, which contribute significantly to decreased exercise capacity, decreased health status, reduced health related quality of life (HRQL), more utilisation of health care resources and increased mortality [3-5].

### The impact of COPD exacerbations

The definition of COPD by the Global Initiative for Chronic Obstructive Lung Disease (GOLD) explicitly mentions COPD exacerbations as an enormous burden for patients [1] and health care systems [6]. Exacerbations are defined as “an event in the natural course of the disease characterised by an increase in dyspnoea, cough and/or sputum beyond normal day-to-day variations. The onset is seemingly acute and may require a change in regular medication or hospitalisation” [1,7]. They are mostly precipitated by an infectious systemic inflammation of the upper respiratory tract and the tracheobronchial tree [1].

Since exacerbations are a significant cause of morbidity (e.g. acute muscle deconditioning and muscle weakness), hospital admissions, impaired health status, impaired quality of life and even death in patients [8-10], prevention is indicated. A relatively small percentage of patients (10%) experiencing frequent exacerbations account for over 70% of all medical costs due to COPD [6]. A study of Pitta et al. (2006) showed that COPD patients tend to be severely inactive during and after an exacerbation; which is worrying since there seems to be a strong association between physical inactivity in patients who recently exacerbated and an elevated risk of (re)hospitalisation due to a COPD exacerbation [11,12]. It appears that patients with recurrent exacerbations show a more rapid decline in their physical activity level than stable patients and their functional capacity gradually decreases faster over time [11]. Besides, their more pronounced skeletal muscle weakness and decreased six-minute walk distance (6MWD, a measure of functional exercise capacity), are risk factors for future exacerbations and higher mortality [9,13]. Consequently, unstable patients, who frequently experience exacerbations, enter a downward spiral of inactivity and exacerbations. Hence, adequate management of exacerbation (prevention) in patients is considered worldwide as one of the main goals in controlling COPD [1].

Although medical treatment modalities for COPD have improved, there is still no pharmacological therapy available that reduces the progression of the disease [4]. Though, patients with COPD, irrespective of disease stage, have shown to benefit from exercise programmes [12] resulting in improved exercise performance and health status [4,5,14-17].

### Physical exercise training in COPD

Evidence, to support the biological plausibility of the positive effects of physical exercise training on COPD, points towards longer high-intensity exercise training programmes. High-intensity exercise training has shown to increase exercise tolerance [18,19]. Firstly, exercise training improves muscle oxidative capacity and oxygen recovery kinetics in patients with COPD [20]. Secondly, patients with COPD who experience lactic acidosis during exercise can attain physiologic training responses from a physical exercise training programme [21,22]. Exercise performance can be improved by reducing the ventilatory requirement for a certain activity level. As the bioenergetics of skeletal muscle improve, blood lactate levels are reduced at a given level of exercise; thereby decreasing the amount of non-metabolic carbon dioxide (CO_2_) that is produced by the bicarbonate buffering system [12,21]. Since lactic acid stimulates ventilation, decreasing lactate production during exercise can be very helpful for patients with COPD. Physical exercise training, in addition to optimal bronchodilatation, can reduce breathing frequency during exercise and consequently lower the degree of dynamic lung hyperinflation that many patients with severe COPD develop [22,23]. In turn, decreased hyperinflation may mediate improvement in exercise endurance by delaying the attainment of a critically high inspiratory lung volume [22,23]. Moreover, high-intensity exercise training, engendering high levels of blood lactate, are more effective than training work rates eliciting low lactate levels [21]. Although measurable physiological changes may occur within weeks, behavioural changes may require longer time periods and may be the reason that greater effects were shown in long-term exercise programmes [19]. In conclusion, extensive physical exercise training is beneficial in patients with COPD.

### Physical exercise training to reduce COPD exacerbation frequency, duration or severity

Patients with COPD often experience sudden worsening of symptoms, i.e. exacerbations. Previous studies already demonstrated that physical exercise training (the component that has shown to provide the most benefit of pulmonary rehabilitation programmes) [1] has important benefits for patients, such as improved exercise capacity and HRQL [9,15,24]. However, the effect of pulmonary rehabilitation and physical exercise training on the occurrence of exacerbations is less clear [4,19,25,26]. Observational studies demonstrated that patients who perform regular physical activity have a reduced risk of hospital admission due to COPD and decreased all-cause and respiratory mortality [12,27,28], but neither of these outcomes are a substitute for reduction of exacerbation frequency, duration or severity. The factors determining utilisation of health care resources in patients with COPD are poorly understood [29]. Few studies reported significantly fewer exacerbations after a pulmonary rehabilitation program (including physical exercise training) [30,31]. Reduction of exacerbations may be at least one of the factors explaining the reduction in health care utilisations as reported in observational studies. Moreover, a recent study suggested that an acute bout of exercise resulted in a reduction in sputum proinflammatory cytokines, suggesting some anti-inflammatory effect of exercise in the airways of patients with COPD [32]. Based on these few findings it is plausible to hypothesise that physical exercise training for patients with COPD may result in fewer exacerbations, or at least in less severe exacerbations, meaning exacerbations with a shorter duration or exacerbations without the necessity for hospital admission.

Prevention of exacerbations by means of physical exercise training would fit the prime management goal for COPD [27]. Based on expert’s opinion, it was stated that teaching patients how to recover quickly from an exacerbation will probably minimise the risk for relapse and improve long-term outcome [26]. Exercise training improves recovery in patients with COPD after an acute exacerbation [33]. Also, from previous studies it has been shown that especially *early* pulmonary rehabilitation (including physical therapy) after an acute exacerbation is most likely to result in clinically relevant improvements in functional exercise capacity and health-related quality of life [5,16]. Puhan et al. (2011) found in nine small trials of moderate methodological quality that effects of pulmonary rehabilitation programmes immediately after an acute COPD exacerbation were visible when at least physical exercise was included [24]. However, they also concluded that more studies are needed to further investigate the role of pulmonary rehabilitation after an acute exacerbation and its potential to reduce costs [24,34].

Evidence based physical exercise training and advice on physical activity can be delivered by physical therapists that follow evidence based guidelines for physical therapy in COPD patients, such as the guideline COPD developed by the Royal Dutch Society for Physical Therapy (KNGF) [35]. This study protocol hypothesis that early protocol-directed physical therapy for patients with COPD may reduce COPD exacerbation frequency, duration or severity.

## Methods/Design

### Study aim

The aim of this study is to assess the clinical effectiveness, cost-effectiveness and cost-utility of early protocol-directed physical therapy for patients with COPD on exacerbations (frequency, duration and severity), health status and quality of life in patients who have recently experienced a COPD exacerbation.

### Study design

**Figure 1:**
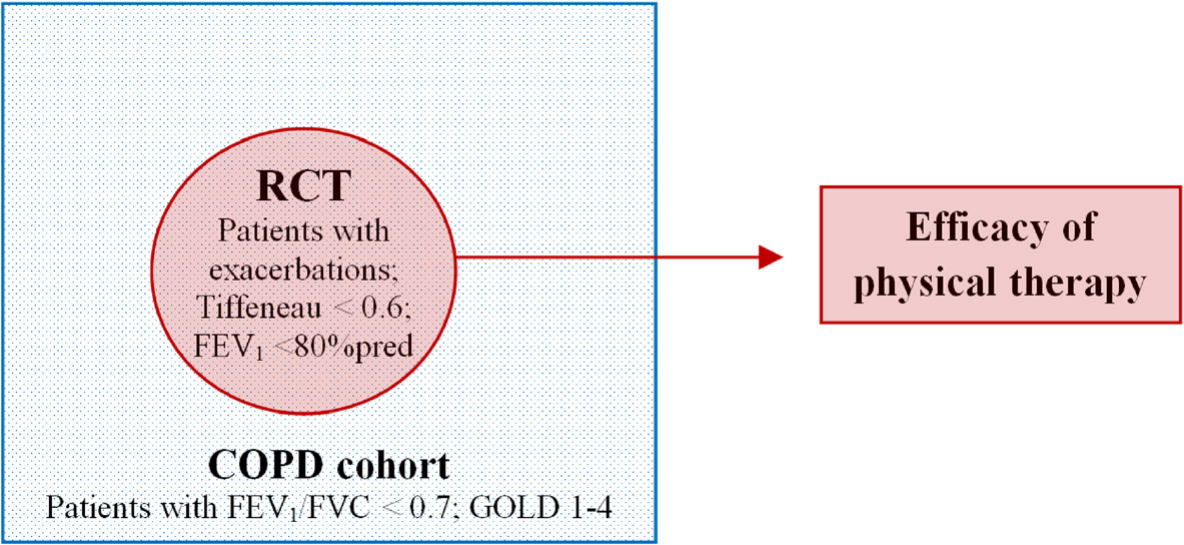
**Framework of the study: a cohort-nested, prospective, randomised controlled trial.** Definition of abbreviations: COPD = Chronic Obstructive Pulmonary Disease; RCT = Randomised Controlled Trial; Tiffeneau < 0.6 = Tiffeneau index (FEV_1_/VC) < 0.6*; FEV_1_ = Forced Expiratory Volume in one second*; FVC = Forced Vital Capacity*; GOLD I = mild COPD, FEV_1_/FVC < 0.7 and FEV_1_ ≥ 80% of predicted*; GOLD II = moderate COPD, FEV_1_/FVC < 0.7 and 50% ≤ FEV_1_ < 80% of predicted*; GOLD III = severe COPD, FEV_1_/FVC < 0.7 and 30% ≤ FEV_1_ < 50% of predicted*; GOLD IV = very severe COPD, FEV_1_/FVC < 0.7 and FEV_1_ < 30% of predicted *or* FEV_1_ < 50% of predicted* plus chronic respiratory failure. *All lung functions are post-bronchodilator values.

A cohort-nested, prospective, randomised controlled trial (cohort-nested RCT) will be conducted. This means that a RCT will be embedded within a COPD cohort (Figure 1). The cohort will consist of COPD patients who receive usual multidisciplinary COPD care and guideline-directed physical therapy (gdPT). The cohort will serve as an optimal recruitment population for the RCT to show the surpassing importance of physical therapy interventions on exacerbations. Therefore, a RCT that holds a large contrast of protocol-directed physical therapy (pdPT) versus sham treatment (ST) will be constructed. This means that when patients from the COPD cohort report an exacerbation, with an occurrence no longer than 56 days ago (a trade-off between threshold values of 35–91 days in which the majority of exacerbations have returned to baseline [36]), they are either randomised to the experimental group (protocol-directed physical therapy (pdPT)) or the control group (sham-treatment (ST)). This RCT will be used to study effectiveness of physical therapy in addition to an economic evaluation.

The ethics committee of Maastricht University has approved the study protocol, procedures and informed consent (NL28718.068.09).

### Study population

**Figure 2:**
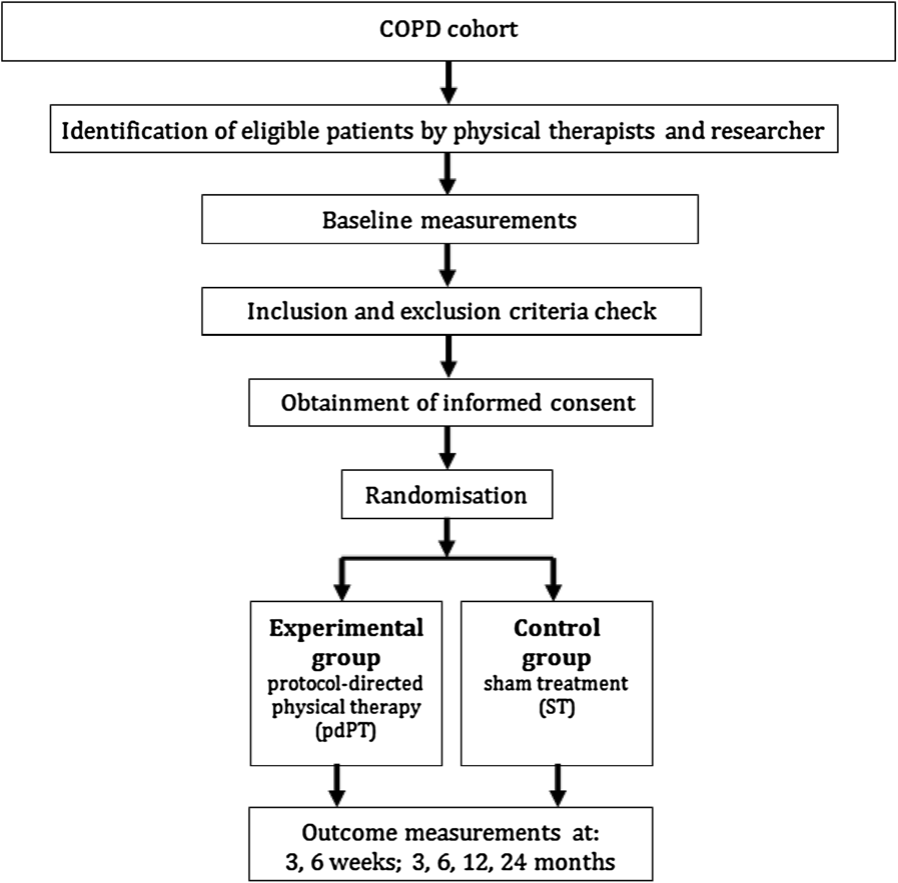
**Flowchart of the RCT.** Definition of abbreviations: COPD = chronic obstructive pulmonary disease; pdPT = protocol-directed physical therapy; ST = sham-treatment, including no or very low-intensity exercise training.

The study population consists of patients who are treated by COPD-specialised physical therapists, after referral by a general practitioner (GP) or pulmonologist. Physical therapy practices, GPs and pulmonologists, in southern districts of the Netherlands, who are willing to participate in the study, will be recruited. Once a patient is referred to a participating physical therapy practice (with post-bronchodilator FEV_1_/FVC < 0.7, GOLD 1–4), the patient will be asked to participate in the COPD cohort study. Patients in the cohort are monitored for health outcomes and exacerbation occurrence. Participants for the RCT will be recruited within the cohort by physical therapists, as soon a patient suffers from an exacerbation. Additionally, advertisements in local papers in southern regions of the Netherlands are used to reach potential COPD patients for the RCT who were missed out on inclusion with the former recruitment method. Those patients who are reached through advertising and presenting with an exacerbation will be recruited for the RCT by the researcher, while the mechanisms for alert and continuous trial recruitment are automatically organized within the cohort. By means of this unique cohort-nested RCT design (Figure 1), the likelihood that patients with a COPD exacerbation will be picked up is higher. A flowchart of the RCT is presented in Figure 2.

The following inclusion criteria will be checked during the eligibility screening of patients for the RCT: a GP/pulmonologist diagnosed COPD in GOLD stage 2, 3 or 4 (supported by a post-bronchodilator FEV_1_/FVC < 0.7 and FEV_1_ < 80% of predicted); eligible for reimbursement by health insurance companies for physical therapy (post-bronchodilator Tiffeneau-index < 0.6); experienced an COPD exacerbations in the past 56 days (defined as: unscheduled visit to their GP/pulmonologist or hospitalisation and possibly receiving a course of antibiotics and/or prednisone); having an adequate and optimal medication (inhalation) regimen by their referring physician; willing to sign informed consent before randomisation; competent enough to speak and understand the Dutch language; above 18 years of age.

Patients who meet the following criteria will be excluded from the RCT: suffering from significant exercise limitations or comorbidities that would prevent a patient from following the required intervention in this study; and expected to be lost for follow-up (for example because of a planned change of residency).

All patients entering the cohort have to give informed consent on the following two levels, whereas patients entering the RCT have to give informed consent on three levels: (1) oral consent to exchange patient information between physical therapists and other related health care providers (usual physical therapy procedure); (2) oral consent to participation in the COPD cohort (registration and use of data for research purposes); and (3) written consent to participate in the RCT, after experiencing an acute exacerbation.

### Size of the study population

For the cohort study 300 patients with COPD will be recruited. This number is based on the calculated sample size for the RCT part of the study. The sample size calculation for this RCT is based on the identification of a minimal relevant clinical difference in exacerbation frequency between the experimental and the control group. The difference in exacerbation rate is expected to be 22% (based on exacerbation rates found in previous studies with rehabilitation programmes) [37,38]. The probability that the study will detect a treatment difference is 80% at a two-sided 5% significance level. This is based on the assumption that the intervention period will be 12 months, the follow-up period will be 24 months and the ratio control subjects to intervention subjects is 1:1. A sample size calculation for comparing event rates between two independent groups is used, resulting in 79 participants per group [39,40]. Assuming outcome data will be analysed prospectively and considering a drop-out rate of 20%, 100 patients per group will be needed in this two treatment parallel-design study.

### Randomisation and blinding

After taking baseline measurements and eligibility screening within 56 days after the start of the past exacerbation, informed consent is obtained and stratified block randomisation will take place (Figure 2). Pre-stratification will be applied for exacerbation severity and GOLD stage (six levels in total), since both factors are suspected to influence treatment outcome. Therewith, the influence of selection bias for severity of the disease on group configuration or comparability between groups may be minimized. The patients will be randomly assigned to the experimental group or the control group in a 1:1 ratio. The concealed randomisation procedure will be performed by a blinded, independent research assistant using a computerised system. A block-randomisation will be used with blocks of size four, six or eight, with random block sequences. A computerized system will refer the patients to a group, after which a blinded, independent research assistant will notify the treating physical therapist after receiving instructions on treatment group allocation through computer generated random number tables.

In this RCT full blinding of the patients and physical therapists is not feasible. However, Hawthorne effects are non-differential, since patients in the experimental group as well as patients in the control group will get attention from their physical therapist. Both groups receive periodic questionnaires and measurement sessions, avoiding non-random effect optimisation. Several measures are taken to avoid bias due to blinding issues. Although patients will be aware of the existence of two treatment arms, they are not informed about the exact content of the other treatment arm (e.g. determining intensity) to prevent influence on outcomes. The control group will be assessed at the same time intervals as the experimental group by trained physical therapists that do not assess the patients in the experimental group at the same time. The researcher is fully blinded, because all outcome measures are quantified by the patients and physical therapists. Physical therapists are blinded for a number of outcomes measurements (questionnaires CCQ, CRQ-SR, GPE, EQ-5D, DS14 and physical activity level), since part of the data that are provided by the patient alone and will be captured through an electronic patient record system. Moreover, a number of important objective outcomes (exacerbation frequency, duration and severity, mechanically assessed physical activity level and cost diaries) are less liable to patient manipulation in order to please their physical therapist, as these outcomes are directly collected by the researcher.

### Cohort

All participants in the cohort receive usual multidisciplinary COPD care including guideline-directed physical therapy. In the Netherlands usual COPD care is given by a pulmonologist and/or a GP. This usual COPD care entails lung function testing, medication prescription for COPD and counselling, including education about the disease, symptoms and risks and tailor-made advice, which basically comes down to two major aspects; stop smoking and increase physical activity in everyday life [41]. In the light of the latter, all patients in the cohort are referred for usual physical therapy and physical activity advice. Usual physical therapy in the Netherlands is guideline-directed physical therapy (gdPT), provided by registered physical therapists and based on the KNFG physical therapy guidelines for COPD [35]. These guidelines provides assistance for applying physical therapy intervention (diagnosis and treatment) in COPD patients coping with impairments in mucus clearance, pulmonary function, muscle function and exercise capacity, and with limited physical activities due to dyspnoea or exercise intolerance. In the guidelines the effectiveness of several treatment modalities (specifically exercise training, breathing exercises, peripheral and respiratory muscle training) was reported and evidence based recommendations for the application of these modalities in physical therapy programmes were made. Moreover, disease management strategies are integrated. Short term goals incorporate improvement of patient’s knowledge, self-management and confidence to accomplish activities. Medium term goals are relief of dyspnoea, improvement of impaired airway (mucus) clearance, and improving or retaining exercise performance and physical activity in everyday life [35]. Long term goals entail improvement or preservation of disease related quality of life. Physical therapists are free to compile a patient-centred program at one’s professional discretion, within the limitations of the guideline. Hence, frequency of the guideline-directed physical therapy for COPD in the Netherlands varies; patients visit their physical therapist generally one to three times a week, for one hour, ranging from one-time consultation to multiple consultations during 3–12 months, depending on disease severity (based on usual care given in participating physical therapy practices) (Table 1).

**Table 1:** Contrasts between RCT experimental and control group and cohort

	***Intervention***	***Content***	***Intensity***	***Frequency***	***Start***
Cohort	**Guideline-directed physical therapy (gdPT):**	One or more of:	Ranging on the full intensity scale	On average:	
		Exercise training, peripheral muscle strength training, respiratory muscle training, breathing exercises, electrical muscle stimulation, physical activity in daily life (homework)		30 minutes to 1 hour	
	A programme made by individual PTs, within the limitations of the KNFG physical therapy guideline for COPD (usual care)				
				1 to 3 times a week	
				During 3 months to multiple years	
RCT experimental group	**Protocol-directed physical therapy (pdPT):**	Both exercise training and peripheral muscle strength training;	Endurance/ interval training ≥ 60% of (sub) maximum, muscle strength training ≥ 80% of maximum, always: borg-scale ≥5	Minimal:	*Early* PT: starting within 56 days after an acute exacerbation
				1 hour	
	A complete programme by protocol, according to the KNGF physical therapy guideline for COPD, but with strict conditions			Twice a week	
		When indicated: respiratory muscle training, breathing exercises, electrical muscle stimulation		During 12 months	
		Always: physical activity in daily life (homework)			
RCT control group	**Sham-treatment (ST):**	One-time consultation: advice to be physically active in daily life			
	No physical therapy				
	*Or*				
				Maximal:	
	Very low-intensity exercise training	Exercise training only	Endurance/ interval training ≤ 15% of (sub) maximum or borg-scale ≤2	30 minutes	
				Once a week	
				During 12 months	

Definition of abbreviations: RCT = randomised controlled trail; COPD = chronic obstructive pulmonary disease; KNGF = the Dutch Society for Physical Therapy.

### Randomised controlled trial

As patients enter the RCT, after experiencing a COPD exacerbation, they may be assigned to either the experimental group or control group. Contrasts between the experimental group, control group and the cohort, based on content, intensity, frequency and timing, are displayed in Table 1.

#### *Experimental group*

Patients in the experimental group receive protocol-directed physical therapy (pdPT). The content of the pdPT is based on the on the KNGF physical therapy guidelines for COPD, like the guideline-directed physical therapy in the cohort [35]. However, in the RCT the physical therapy follows a strict protocol. The therapy specifically concerns *early* physical therapy, starting within 56 days after an acute COPD exacerbation. Patients in the experimental group receive a programme of one hour, twice a week for one year. The programme includes high-intensity exercise training, which entails endurance and/or interval training with an intensity of 60% or higher of (sub) maximum physical exertion, based on the results of a maximal cardiopulmonary exercise test (CPET) and six-minute walk test. In addition, ratings of perceived exertion and dyspnoea of five or higher on the modified Borg-scale (0–10) are used to tailor exercise intensity [42]. Peripheral muscle strength training is provided (a programme combining upper and lower extremities) with an intensity of 80% or higher of maximum physical exertion, based on the results of one-repetition maximum tests or handheld dynamometer measurements. Again, ratings of perceived exertion and dyspnoea of five or higher on the modified Borg-scale are used to adjust exercise intensity [9]. When indicated, the programme includes respiratory muscle training, breathing exercises and electrical muscle stimulation. In addition, much emphasis is given to the assessment and treatment of physical inactivity in daily life. Patients are asked to increase their total physical activity on their own. At least 30 minutes of moderately intense physical activity on at least five days a week is the current recommended level. All visits for treatment and all advice for home training are part of standard procedure, following the KNGF guideline physical therapy for COPD and the Dutch Standard for Healthy Exercise (NNGB) [35,43]. An additional file shows the intervention in more detail according to a framework based on the International Classification of Functioning, Disability and Health (ICF) [see Additional file 1] [44].

#### *Control group*

Patients in the control group receive sham-treatment (ST), which entails no or very low-intensity exercise training, for one year. The latter applies only if the participant in the control group insists on training in a physical therapy practice. The very low-intensity exercise training is limited to a maximum of 30 minutes once a week, with an intensity of 15% or lower of (sub) maximum physical exertion, based on the results of a maximal CPET and six-minute walk test. In addition, ratings of perceived exertion and dyspnoea of two or lower on the modified Borg-scale (0–10) are used to tailor exercise intensity [42]. There will be no further peripheral muscle strength training, respiratory muscle training, breathing exercises nor electrical muscle stimulation [35]. Furthermore, patients will be advised to do at least 30 minutes moderately intense physical activity on their own for at least five days a week according to the physical activity norm [43].

### Physical therapists

All interventions are carried out in primary care physical therapy practices. Registered physical therapists, experienced in COPD care, who are willing to participate in the study, will be recruited through local physical therapy networks in the Netherlands that already have mapped the specialised skills of these health professionals and registered who treats a sufficient number of patients with COPD (with a minimum of 5–10 per week).

Therapists will be invited to attend information/training sessions given by the research group and related COPD experts. Information will be given about the aim and content of the cohort and RCT along with information about the necessary clinical measurements. Lectures will be given on the latest information on COPD and updates on the current COPD guidelines and on counselling (in line with the Dutch college of general practitioners (NHG) standard COPD). Furthermore, a COPD master class will be given to the network of participating physical therapists in cooperation with the Dutch Paramedical Institute (NPi).

### Data collection

Participants will be monitored by physical therapists with the help of a high quality electronic patient record system that is COPD-specific and serves simultaneously as a research database for the experimental group as well as for the control group. Moreover, the system will be used by physical therapists as guidance for treatment of the experimental group, since the KNGF guidelines physical therapy for COPD is fully incorporated within this system (for example: an alert will be visible on the computer screen when a patient’s performance is below 80% of the predicted peripheral muscle strength or when FEV_1_ <50% and Medical Research Council dyspnoea score (MRC) ≥2 an advice to initiate multidisciplinary evaluation and rehabilitation is given).

Primary and secondary study-specific outcome measures will be assessed with the help of the COPD-specific record system. In addition, the system will contain information about the content of physical therapy, the duration of physical therapy, the total amount of physical therapy sessions, the duration per session, and the adherence to physical therapy.

#### *Primary outcome measure*

The primary outcome measure will be exacerbation frequency, calculated as the number of COPD exacerbations experienced by the patient in a post exacerbation period of two years. A COPD exacerbation will be identified as a sustained worsening of the patient’s condition occurs from the stable state and beyond normal day-to-day variations that is acute in onset and may warrant additional treatment [45]. A recurrent exacerbation will be defined as a subsequent occurrence; exacerbations are assumed to be independent of each other. The follow-up period is twelve months (twenty-four months for the long-term outcome measurement) to overcome time confounding, since exacerbation frequency is seasonal and particularly related to influenza and other viral epidemics [45].

In the cohort, exacerbations will be identified by means of an event based approach, whereas in the RCT exacerbations will be identified by means of an event based approach (health care contact) and symptom based approach (clear increase of respiratory symptoms). Usual assessment of exacerbations is done by recording health care contacts. However, there are many underreported exacerbations in COPD patients when using this event based method [46]. On average, these underreported exacerbations have similar severities to reported exacerbations [45]. Therefore, increase of respiratory symptoms will also be recorded in the RCT. When measuring the appearance of exacerbations with these two methods, this trial is able to compare event based and symptoms based methods in relation to the primary and some of the secondary outcome measures.

#### *1. Event based approach*

The onset of an exacerbation will be the first day of an unscheduled health care contact with a GP/pulmonologist due to a COPD exacerbation and the start of additional medication intake or the first day of an unscheduled hospitalisation or emergency visit to the hospital due to an exacerbation. The exacerbation lasts until the last day of this extra medication intake or until the last day of hospital admission [45]. To identify these events, physical therapists will monitor patients’ reasons for absenteeism on physical therapy appointments and relapse in treatment (as an exacerbation might be the reason). In addition, records of health care contacts and additional medication use due to COPD are registered on patients’ prospective daily diary cards. Overall, good compliance to register for instance symptoms, can be achieved using daily diaries in COPD [47].

#### *2. Symptom based approach*

Respiratory symptoms will be monitored using prospective daily diary cards. In these diary cards patients have to report, according to Anthonisen et al. (1987), whether their major symptoms (breathlessness, sputum production, sputum colour) and minor symptoms (cough, wheeze, running nose, score throat, and fever (>38.5°C) were beyond normal [7]. Prospective diary card assessments are best recorded as changes from an agreed baseline, rather than absolute symptom severities. Comparable to the COPE-II study by Effing et al. (2009), at inclusion all patients will receive a ‘what is normal for me’ card, which describes their individual levels of major symptoms at baseline [48]. When patients experience no deterioration of any of the symptoms listed on the diary, they should check ‘no change in symptoms’. When patients experience deterioration, they should check ‘yes’ and report on all symptoms in the diary whether the level of each symptom was ‘normal’, ‘slightly increased’, or ‘clearly increased’.

The onset of an exacerbation will be defined as the first day of at least two consecutive days at which the patient checks ‘clear increase’ from baseline in two major symptoms or one major and one minor symptom. The day that an exacerbation is resolved will be defined as the first day of: (1) three successive days that the patient has returned to his normal health state; or (2) seven consecutive days on which patients continuously reported no or only a ‘slight increase’ in symptoms, compared to baseline, with no fever or change in sputum colour [7,48,49]. Besides the daily diary card, symptoms during COPD exacerbations will also be recorded by the physical therapists, in order to receive additional information about the number and type of perceived symptoms and to add missing information to the daily diary cards in cooperation with the patient.

#### *Secondary outcome measures*

Exacerbation duration is defined as the duration of the medical intervention per occurrence and the number of days with clear increase of respiratory symptoms. For the event based approach: the duration of an exacerbation will be equal to the time of the medical intervention [45]. For the symptom based approach: the duration of an exacerbation will be equal to the time between onset and resolution of exacerbation based on the above mentioned definitions [7,48,49].

Furthermore, a distinction is made between various levels of exacerbation severity. For the event based approach, a scale for exacerbation severity is proposed, which distinguishes two levels of exacerbation severity: (1) mild/moderate and (2) severe. Level one exacerbations can be treated at home by means of a health care contact with a GP/pulmonologist and the start of additional (temporarily) medication intake (e.g. corticosteroids or antibiotics). Level two exacerbations require hospitalisation or an emergency visit to the hospital [45,46]. For the symptom based approach: the severity of an exacerbation day will be calculated with help of symptom scores. The major symptoms are scored as: normal = 0; small increase = 1; or clear increase = 2. The minor symptoms are scored 0, 0.5 and 1, respectively. Sputum colour will be scored as: normal = 0, different from normal = 2; and fever: no = 0, yes = 1. Adding all scores, results in a daily symptom score ranging from 0–11 points. When patients are admitted to the hospital for their COPD, a daily score of the maximum 11 points will be assigned [7,48].

Generic health related quality of life (HRQL) of the patients will be assessed by means of the Euro-Qol (EQ-5D-3L). Disease-specific HRQL will be assessed with the Clinical COPD Questionnaire (CCQ) and the Chronic Respiratory Questionnaire – self reported (CRQ-SR). In addition, negative affectivity, social inhibition, and type D personality will be assessed by means of the DS14 questionnaire.

The level of dyspnoea (MRC), effective mucus clearance, physical activity in daily life and motivation will be assessed by standardised questions from the KNGF guidelines [35]. Furthermore, to estimate the patients’ perceived benefit of the physical therapy, the Global Perceived Effect (GPE) will be scored by patients on a 9-point scale [35]. Objective assessment of physical activity in daily life will be done by means of an accelerometer-based activity monitor (Dynaport MoveMonitor) [50].

Height, weight, Body Mass Index (BMI) and Fat Free Mass (FFM) will be measured. The peripheral muscle strength of the patient will be measured as maximal voluntary isometric contraction (MVIC) of the dominant hand, shoulder abduction and knee extension (m. quadriceps), tested in standardised positions by means of the break method by a handheld dynamometer [42,51]. Functional exercise capacity is measured by the six-minute walk test (6MWT), in accordance with the guidelines by the American Thoracic Society [52]. If a straight floor-walking course of 30 metres is not available in the physical therapy practice, a course with a distance of 10 metres is the required minimum [35]. Outcome is measured by total walking distance in metres (6MWD) and in percentage of the predicted value. Reference equations by Hill et al. (2011) will be applied to calculate the predicted value if a straight 30 metre walking course was used, whereas reference equations for a straight 10 metre walking course may be necessary to develop within this study, as no reference equations exist for this frequently used test layout [53]. During the walk test, perceived fatigue and dyspnoea will be measured on a modified Borg scale ranging from zero (nothing at all) to ten (very, very severe) [42]. Transcutaneous oxygen saturation and pulse rate will be measured with a finger pulse oximeter (Onyx 9500) [52]. The modified Get Up and Go test (mGUG) is used to measure basic functional mobility. The outcome is the time it takes a subject to stand up from a chair and walk a distance of 10 metres [54]. After the mGUG, perceived exertion is measured with the modified Borg scale.

Comorbidities of all participants will be recorded thoroughly, as well as smoking history (pack years and cessation moment) and physical therapy compliance (scored by the therapist as insufficient, moderate or good and scored whether comorbidity has any influence on this compliance), as being important potential confounders in this study. Number and type of adverse events will be registered during the one-year treatment period. After randomisation, patients are discouraged to participate in interventions other than those in the study (like hydrotherapy or drug studies) that may influence the outcome measures. When they still decide to participate in other interventions, these interventions will be registered during the study period.

Finally direct and indirect costs will be assessed with three-monthly retrospective questionnaires, including questions about health care contacts, medications use, residential status, occupational status, domestic care service and use of medical aids. The economic evaluation will be analysed from a societal perspective. This means that the most appropriate set of costs captured from the data, regardless of where the costs or benefits occur, will be applied.

#### *Planning of outcome assessment*

In all eligible patients, entering the cohort and the RCT, baseline assessment will be performed. In the baseline assessment, demographic variables, anthropometric data, healthcare/medical variables, lifestyle factors, health related quality of life and clinical status will be recorded of the participants.

The lung function of the patients is already assessed by the GP/pulmonologist or assistant, by means of pulmonary function tests, as part of standard procedure in patients with COPD. Forced expiratory volume in one second (FEV_1_ (L)), forced vital capacity (FVC (L), the FEV_1_/FVC ratio and Tiffeneau-index (FEV_1_/VC) are measured by means of post-bronchodilator spirometry and are known to the physical therapist in order to construct a tailored-made programme adjusted to the severity of the pulmonary component of the disease. The clinical status, maximal exercise capacity and physiological restrictions of the patients should in most cases have been assessed by a maximal CPET and communicated to the physical therapist in order to enable the physical therapist to construct a safe and tailored-made programme for the patient. If the clinical status of the patient in the RCT is not yet assessed in the hospital or rehabilitation clinic, the treating physical therapist will be asked to refer the patient for a maximal CPET to a sports medicine physician, who is contacted by the researcher.

**Figure 3:**
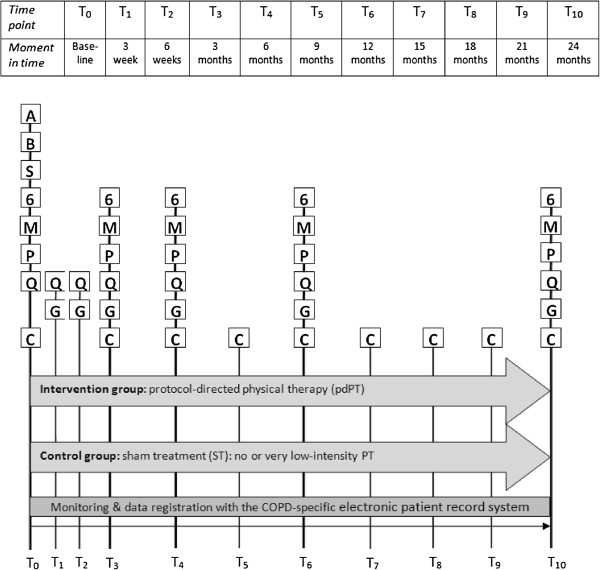
**Planning of outcome measurements in the RCT.** The diary cards are not included in the Figure, but they are used by the patient every day of every month until T_6_. In the cohort, the baseline measurement is followed by the same measurements as on T_3_ in the RCT and are repeated every three months for at least twelve consecutive months. Definition of abbreviations: T_0_ = baseline measurement; T_1_ – T_10_ = consecutive measurements in time after the baseline measurement; A = Anthropometric measures; B = Bicycle test results (maximal cardiopulmonary exercise test (CPET)); S = Spirometry results; 6 = 6MWT + Borg score and mGUG + Borg; M = peripheral muscle strength; P = physical activity in daily life with accelerometer; Q = CCQ*, CRQ-SR, EQ-5D*, DS14, MRC and level of effective mucus clearance, level of motivation, physical activity, physical therapy compliance*; C = questionnaire to assess direct and indirect costs; and G = Global Perceived Effect*. Measurement occasions are explained in the Table above the Figure. *The only measurements on T_1_ and T_2_.

After the baseline visit, participants in the cohort will be assessed every three months for 12 months or longer. Participants from both the experimental and the control group in the RCT will be measured at three and six weeks, and three, six, twelve and twenty-four months during follow-up visits at the physical therapy practice for various outcome measures (Figure 3).

In the RCT, prospective daily diary cards are distributed every month for twenty-four consecutive months and are returned by the patient at the end of every month. The retrospective questionnaire on the use of health care is send to the patient once every three months for twenty-four consecutive months.

### Data analysis

#### *Clinical effectiveness*

The descriptive characteristics are presented quantitatively as mean (±standard deviation) or median (5^th^ - 95^th^ percentile), depending on the data distribution, whenever the variable is continuous or as a percentage whenever the variable is dichotomous or categorical. Data will be analysed with SPSS-19. Assumptions accompanying the following statistical methods will be checked and all data analyses will be applied by a blinded analyst.

Data from the RCT will be analysed to examine intervention main effect on the counts for the primary outcome (exacerbation frequency) and the secondary outcomes. All exacerbations from each patient will be used as the dependent outcome to evaluate the effect of physical therapy at the end of the intervention (12 months) and follow-up (24 months). Measurement moments for secondary outcomes are baseline, three and six weeks, three, six, twelve and twenty-four weeks. The effects of the variable of primary interest, physical therapy (pdPT), will be analysed in a generalized linear model (GLM) for group (intervention vs. control) analysis of covariance with adapted regression for count data distribution. Analyses of secondary outcomes will be done with linear regression analysis and generalized linear models. Since exacerbation outcome in the RCT is measured with two methods, analyses will be based on the event-based approach, on the symptom-based approach and on a combination of both. Potential confounding by variables like, age, gender, GOLD stage, smoking, physical activity in daily life, exacerbation history, severity of latest exacerbation, and comorbidity, will be controlled for. Results from a ‘per-protocol’ and an ‘intention-to-treat’ principle will be compared. At a minimal statistical power of 80%, p-values smaller than 0.05 will be considered as significant.

#### *Economic evaluation*

From the RCT data cost-effectiveness and a cost-utility of physical therapy in COPD patients experiencing exacerbations compared to usual care will be established using a one and two year time horizon.

A cost-effectiveness analysis will be performed, weighing incremental costs against the mean incremental effect in terms of quality of life based. For assessing patient outcome, the score of the disease specific quality of life measures CCQ and CRQ-SR will be used at the 12 months follow up moment. For this purpose, a societal perspective will be administered, indicating the relevance of direct medical, direct non-medical and indirect costs, as adding measurement of indirect costs will provide a more comprehensive picture of the burden of exacerbations [55]. For defining the content of care ‘consumed’, out of pocket costs, lost productivity costs and direct non-medical costs for each patient in the RCT, resource use will be measured using patient questionnaires. These frequently used retrospective questionnaires will cover a three months retrospective period. Moreover, patients’ daily diary cards will be assessed, to incorporate each exacerbation experienced by a patient that involves ‘consumed’ care. The costs of the experimental physical therapy will be assessed using micro-costing and time-and-motion techniques in several participating practices and patients’ dossiers of the physical therapist will be assessed.

For the cost-utility analysis, quality adjusted life year (QALY) will be calculated based on the EuroQol questionnaire (EQ-5D-3L). The EQ-5D-3L is a generic quality instrument that will make it able to calculate utilities at each outcome measurement (on a 12 months’ and 24 months’ basis). Thus, societal cost will be used to calculate the incremental cost-effectiveness based on CCQ and CRQ-SR and to calculate the incremental cost-utility based on QALYs. Besides, the cost of the physical therapy intervention will be assessed against the incidence of exacerbation in both groups within the RCT.

### Project time frame

The project in total will take five years. During the first three years of the project, physical therapy practices and COPD patients will be recruited. Patients in the cohort will be followed at least until the end of the project. Patients included in the RCT will be followed for two years by means of the COPD-specific electronic patient record system, while the treatment will be one year. The last year of the project is reserved for the processing and analysis of data, as well as the publication of results.

## Discussion

In this article the protocol of a cohort-nested randomised controlled trial is presented, concerning a study about the effect and cost-effectiveness of standardised physical therapy as a supportive measure to prevent or postpone future exacerbations, to shorten the duration or to decrease the severity of future exacerbations in COPD patients experiencing acute exacerbations. The focus on prevention of exacerbations by means of physical therapy fits one of the prime management goals for COPD, which is ‘reducing the frequency of hospitalisations due to exacerbations’ [1]. It is expected that the outcomes of this study will provide useful information about the effects of physical therapy on exacerbations, which may alter the role of physical therapy in managing COPD in the future.

Healthcare utilisation is not an optimal substitute for exacerbation frequency, exacerbation duration and exacerbation severity, depending on many unrelated social factors and comorbidity. It is an outcome in its own right [45]. Therefore, not only will this event based approach be applied in this trial, also a symptom based approach will be used. Both approaches will be used separately and together and their influence on outcome will be compared during statistical analyses.

### Bias and confounding

To assure a high quality treatment according to KNGF guidelines physical therapy for COPD, the participating physical therapists will be trained before the start of the study. It is obvious that physical therapists as well as patients cannot be fully blinded during this study, since both groups are aware of the treatment procedures. Moreover, due to practical reasons physical therapists perform some exercise measurements instead of the researcher. This can lead to subjective interpretations of the research findings. Therefore, participating physical therapists will be instructed to make correct use of the measurement instruments for standardisation. To minimise social desirability answering, patients are told that there are no right or wrong answers before filling out questionnaires. In order to minimize differential Hawthorne effects, all patients from the cohort, the intervention group and the control group in the RCT will be monitored very precisely in a standardised way on first signs of exacerbations and other outcome measurements. To minimise bias, research findings will be analysed by a blinded researcher.

### Potential problems

Potential problems regarding the execution of this study are twofold. Because COPD is a complex disease the KNGF guidelines for physical therapy for COPD patients requires a lot of effort from participating physical therapists regarding patient assessment, apart from the general KNGF Guidelines on Reporting in Physical Therapy. Although the outcome measures in this study are very well connecting to the requirements of these guidelines, complete registration remains difficult and costs time, so problems may be expected regarding the full registration of COPD patients in this study.

Besides, patients who enter the RCT may be assigned to the control group, which may come with some problems. To include patients in the control group means physical therapists are only allowed to offer no or very low-intensity exercise training. Due to the lower frequency of treatments, participation in the RCT may be financially unattractive to the physical therapy practice and physical therapists. Moreover, there is a chance that patients in the intervention group of the RCT are doing much better compared to the control group.

## Abbreviations

COPD: Chronic obstructive pulmonary disease; DO-IT: ‘Designing optimal interventions in physical therapy’, the research programme in which this study is embedded; WHO: World Health Organisation; GOLD: The Global Initiative for Chronic Obstructive Lung Disease; FEV1: Forced expiratory volume in one second; FVC: Forced vital capacity; VC: Vital capacity; KNGF: The royal Dutch Society for Physical Therapy; NHG: The Dutch college of general practitioners; RCT: Randomised controlled trial; gdPT: Guideline-directed physical therapy; pdPT: Protocol-directed physical therapy; ST: Sham-treatment, including no or very low-intensity exercise training; GP: General practitioner; NPi: The Dutch Paramedical Institute; CPET: Maximal cardiopulmonary exercise test; NNGB: The Dutch Standard for Healthy Exercise; ICF: The International Classification of Functioning, Disability and Health; HRQL: Health related quality of life; CCQ: Clinical COPD Questionnaire; CRQ-SR: Chronic Respiratory Questionnaire, self-reported; EQ-5D: Euro-Qol, 5 domains; DS14: Questionnaire to measure type D personality: negative affectivity and social inhibition; MRC: Medical Research Council dyspnoea score; GPE: Global perceived effect; BMI: Body mass index; FFM: Fat free mass; MVIC: Maximal voluntary isometric contraction; 6MWT: Six-minute walk test; 6MWD: Six-minute walk distance; mGUG: Modified Get Up and Go test; RM ANOVA: Repeated measures analysis of variance; QALYs: Quality adjusted life years; WMO: The medical research involving human subjects act.

## Competing interests

The authors declare that they have no competing interests.

## Authors’ contributions

EB substantially contributed to the intervention protocols, recruitment and the draft of the manuscript. RdB, EH, GW, GA, HH, RG, SE and OvS were involved in the design of the study, drafting the manuscript and revising it critically. RdB, EH, IM, JM, GW and AF contributed to recruitment of cooperating parties (GP, pulmonologist, physical therapy practices). IM is involved in drafting the manuscript and revising it critically. EB, EH and AF provided information/training sessions and EB and RG provided a COPD master class for participating physical therapists, which enhanced the quality of the intervention in the study. All authors read and approved the final manuscript.

## Pre-publication history

The pre-publication history for this paper can be accessed here:

http://www.biomedcentral.com/1471-2466/14/71/prepub

## Supplementary Material

Additional file 1 **Description of the main goals and content of the protocol-directed physical therapy intervention for a patient with COPD within the cohort-nested RCT, according to a framework based on the International Classification of Functioning, Disability and Health (ICF).** Description: An evidence-based framework for describing goals and content of exercise intervention. The framework that is developed by van der Leeden and colleagues (2013) is a response to the requirements by the CONSORT statement for precise detail of interventions and provides structure for use in research reports [44]. Click here for file
