# Supplementary material for: Exacerbations in patients with chronic obstructive pulmonary disease receiving physical therapy: a cohort-nested randomised controlled trial
Source: BMC Pulm Med. 2014 Apr 26;14:71. doi: 10.1186/1471-2466-14-71 (PMC4108017; doi:10.1186/1471-2466-14-71)
Supplement: Additional file 1 — Description of the main goals and content of the protocol-directed physical therapy intervention for a patient with COPD within the cohort-nested RCT, according to a framework based on the International Classification of Functioning, Disability and Health (ICF). Description: An evidence-based framework for describing goals and content of exercise intervention. The framework that is developed by van der Leeden and colleagues (2013) is a response to the requirements by the CONSORT statement for precise detail of interventions and provides structure for use in research reports [44]. [file 1471-2466-14-71-S1.doc]

Additional file 1: Description of the main goals and content of the protocol-directed physical therapy intervention for a patient with COPD within the cohort-nested RCT, according to a framework based on the International Classification of Functioning, Disability and Health (ICF)[[1]](#footnote-2)

| Intervention protocol |
| --- |
| **Phase 1 (12 months, including individual adjustments to the programme regarding adjustment of treatment goal, type of exercises and intensity at least every 3 months)** |
| **General information:**  Note: This physical therapy intervention is reserved to COPD-specialised physical therapists, meaning registered physical therapists who are experienced in COPD care, who acquired specialised skills through COPD education and who treat a sufficient number of patients with COPD (with a minimum of 5-10 per week).   - Physical therapy is part of respiratory rehabilitation. - The physical therapy starts with history-taking and clinical examination and assessment of exercise performance, respiratory and peripheral muscle function, physical activity and quality of life to determine goals for physical therapy. - After an exacerbation the patients is motivated to resume with the physical therapy sessions as soon as possible. - The intervention is based on the latest Royal Dutch Society for Physical Therapy (KNGF) guideline for physical therapy in patients with COPD (2008). The objective of this guideline is to describe evidence-based physical therapy – with regard to effectiveness, efficiency and tailored care – for COPD patients with impairments in mucus clearance, pulmonary function, peripheral and respiratory muscle function, exercise capacity, and quality of life, and with physical activity limitations in daily life due to dyspnea or exercise intolerance. |
| **Intervention treatment goals:**  The treatment goals[[2]](#footnote-3) for the physical therapy interventions are: (1) to reduce dyspnea; (2) to improve exercise capacity and physical activity; (3) to improve mucus clearance; (4) to improve knowledge, self-management and self-efficacy. In this elaboration of the intervention the main focus of physical therapy is on reducing dyspnoea and improving exercise capacity.  **Above treatment goals according to ICF-classification:**   | **On the level of body functions:** | For the treatment goals, the main focus is based on the most limiting factor of the exercise limitation: | | --- | --- | | **b410 Heart functions** (+ b460 sensations associated with cardiovascular functions) **b440 Respiration functions** (e.g. respiration rate, rhythm, depth) (+ b460 sensations associated with respiratory functions) **b445 Respiratory muscle functions b740 Muscle endurance functions**  **b152 Emotional functions** (e.g. fear of activity, fear of dyspnoea, anxiety, depression) | 1. Cardiocirculatory limitation 2. Ventilatory limitation 3. Oxygen transport limitation 4. Peripheral muscle weakness 5. Psychologic limitation | | **On the level of activities**: |  | | **d410 Changing basic body position**  **d455 Moving around**  **d460 Moving around in different locations**  **d498 Mobility, other specified** (carrying out daily routine, doing housework, dressing) |  |   NB: On the level of functions: if serious weight loss occurs in a patient (>10% in the past half year or 5%> in the past month) or if the patient experiences a COPD exacerbation, multidisciplinary consultation/referral may be indicated. |
| **Exercises:**  The protocol-directed physical therapy programme takes one hour, twice a week for one year. Since it is not possible to fully disentangle the effect of the exercises on the above mentioned levels of body functions, because COPD is a chronic disease with systemic effects, an individual exercise is not assigned specifically to an individual level of body function. However, depending on the most limiting factor(s) in functions: guided endurance training, interval training, muscle strength training, breathing exercises, body positioning, relaxation exercises, education, counselling or a combination will be provided.   - Cardiocirculatory: endurance training - Ventilatory limitation or if oxygen transport in the lungs is disturbed: endurance training versus interval training: If hypoxemic / hypercapnic during exercising, than interval training. If walking on 70% of Watt_max_ for at least ten minutes is possible, than endurance training, if not interval training. - Consequently, if hyperinflation is present, than advises and exercises targeting body position and various breathing exercises (active expiration, slow and deep breathing, pursed lips breathing (PLB), diafragmal respiration). - Consequently, if respiratory muscle weakness is present, than inspiratory muscle training (IMT) - Peripheral muscle strength limitation: endurance / interval training and muscle (resistance) training of both upper and lower extremities and possibly electric muscle stimulation (NMES) in the case of serious decreased muscle strength. - Psychologic limitation: relaxation therapy, education or counselling on physical activity (to enhance compliance and improve self-management skills).[[3]](#footnote-4)   Settings:  Cardiorespiratory endurance / interval exercises:  Frequency: 2 x per week during therapy  Intensity: 60% ≥ of Watt_max_ in maximal cardiopulmonary exercise test (CPET) or maximum walking speed in six-minute walk test and/or a score of 5 ≥ on the modified Borg scale (0-10)  Duration: 20-60 minutes per session (depending on duration of other exercises).  Exercises examples: walking on a treadmill, walking on a cross trainer including arm movements, cycling or rowing on an ergometer.  Muscle strength and muscle endurance capacity:  Frequency: 2 x per week during therapy  Intensity: 80% ≥ of KgF or Nm and/or a score of 5 ≥ on the modified Borg scale (0-10)  Duration: 2 to 5 sets of 8 to 15 repetitions, with 30 seconds of rest between sets.  Main muscle (groups): quadriceps, hamstrings, leg/arm adductors, leg/arm abductors, biceps, triceps, deltoids, pectoralis major.  Exercises examples for upper extremities: lat pull down, upright rowing with a weight or theraband, vertical rowing with a pulley.  Exercises examples for lower extremities: knee extension, leg press, leg abduction/adduction with a pulley, dumbbell squat, sit to stand/chair squat, steps aerobic workout.  Depending on the most limiting factor(s) in activity and participation: education and advice, guided endurance training, interval training and optimising posture, supplemental oxygen, counselling or a combination will be provided.   - Education and advice about risk factors and prognostic factors for COPD, course of the disease, exacerbations. - Education, instruction and stimulation to be physically active in everyday life, apart from the activity in the physical therapy practice (e.g. time aspects, modalities – kind of exercise, frequency, and etcetera), and regarding use of aids. Meanwhile, feedback by means of questionnaires and accelerometer. - Optimising body posture (e.g. during sitting/standing: forward leaning posture and spreading the knees, while keeping the spine relatively straight, placing hands on thighs or knees, and then bracing the upper arms and shoulders for more efficient use of the accessory breathing muscles, during the use of a ‘rollator’ (rolling walker)). - When indicated: The use of supplemental oxygen during exercises (only on medical prescription). The use of short-acting bronchodilators before the therapy starts (only on medical prescription). - When indicated: Specific education and counselling must be addressed or patient must be referred to a psychologist, when a psychological factor (e.g. lifestyle, coping strategies, social context, personality factors, fear of activity or dyspnoea) seems one of the most limiting factor during exercise.[[4]](#footnote-5)   **Home exercises:**  Perform at least 30 minutes of moderately intense physical activity (e.g. walking, cycling) on at least five days a week, according to the physical activity norm. |
| **Phase 2 (12 months – 24 months follow-up period)** |
| - After the 12 month-intervention the patient is free to decide to stop the physical therapy intervention or to continue or adjust the physical therapy programme in consultation with the physical therapist. - The advice to be physically active in everyday life, according to the physical activity norm (perform on at least five days a week physical activity with moderate intensity for at least 30 minutes per activity), applies in every way |

1. van der Leeden M, Staal JB, Beekman E, Hendriks H, Mesters I, de Rooij M, de Vries N, Werkman M, de Graaf-Peters V, de Bie R *et al*: **Development of a framework to describe goals and content of exercise interventions in physical therapy: a mixed method approach including a systematic review**. *Physical Therapy Reviews* 2013:*In press*. [↑](#footnote-ref-2)
2. The global treatment need to be individualised and formulated according to a SMART-criteria. [↑](#footnote-ref-3)
3. For the behavioural component of physical therapy knowledge of behavioural change is necessary. [↑](#footnote-ref-4)
4. For the behavioural component of physical therapy knowledge of behavioural change is necessary. [↑](#footnote-ref-5)
